# Supplementary material for: Effect of Dietary Riboflavin Levels on Reproductive Performance of Pigeon Breeders, and Growth Performance and Carcass Traits of Offspring Squabs
Source: Animals (Basel). 2024 Aug 20;14(16):2414. doi: 10.3390/ani14162414 (PMC11350774; doi:10.3390/ani14162414)
Supplement: Supplementary file 1 [file animals-14-02414-s001.zip › animals-3108861-supplementary.pdf]

Supplementary data

Indicator	Shapiro-Wilks test	*F*-value	*P*-value adjustment	*P*-value actual	Z-value
Initial body weight	0.778	0.363	1.00	0.843	
Final body weight	0.756	0.901	1.00	0.469	
Total feed intake of nursing	0.526	0.081	0.840	0.993	
Laying interval	0.552	1.140	0.373	0.339	
Egg production	0.577	0.329	0.946	0.86	
Egg fertility	0.851	0.733	1.00	0.553	
Egg hatchability	0.000				0.046
Egg weight	0.685	1.563	0.354	0.193	
Egg shape index (vertical diameter)	0.998	0.726	0.907	0.577	
Egg shape index (horizontal diameter)	0.579	0.599	0.913	0.664	
Eggshell color	0.166	0.377	1.00	0.484	
Eggshell strength	0.222	1.145	0.342	0.342	
Egg albumen height	0.423	1.537	0.319	0.203	
Egg yolk color	0.058	4.493	0.017	0.003	
Haugh units	0.661	0.938	1.00	0.447	
Egg yolk weight	0.786	0.866	1.00	0.488	
Eggshell weight	0.215	1.190	0.395	0.323	
Eggshell thickness	0.522	1.584	0.411	0.187	
Carcass weight	0.664	0.261	0.827	0.902	
Eviscerated weight	0.023				0.024
Eviscerated percentage	0.010				0.421
Half-Eviscerated weight	0.086	3.098	0.066	0.018	
Half-Eviscerated percentage	0.037				0.218
Breast muscle weight	0.007				0.015
Breast muscle percentage	0.009			0.002	0.005
Leg muscle weight	0.747	1.255	0.395	0.287	
Leg muscle percentage	0.350	0.290	0.884	0.884	
Body weight 1d	0.007				0.002
Body weight 7d	0.024				0.627
Body weight 14d	0.2	1.09	1	0.364	
Body weight 28d	0.113	1.620	0.466	0.169	
Liver weight	0.004				0.002
Liver index	<0.001				0.001
Spleen weight	<0.001				0.987
Spleen index	<0.001				0.984
Bursa weight	0.012				0.236
Bursa percentage	0.017				0.4
Parental plasma ♀	<0.001				<0.001

Parental plasma ♂	0.005				0.004
Egg yolk	<0.001				<0.001
Squab plasma	<0.001				<0.001
Squab liver riboflavin	<0.001				<0.001
Squab liver FAD	0.010				<0.001
Squab liver FMN	<0.001				<0.001

F-value, *P*-value adjustment, and *P*-value actual were analyzed by ANOVA tests. *Z*-value was the *P*-values of K-W tests.
